# Downregulation of Vascular Hemeoxygenase-1 Leads to Vasculopathy in Systemic Sclerosis

**DOI:** 10.3389/fphys.2022.900631

**Published:** 2022-05-05

**Authors:** Rebecca L Ross, Georgia Mavria, Francesco Del Galdo, Jacobo Elies

**Affiliations:** ^1^ Leeds Institute of Rheumatic and Musculoskeletal Medicine, Faculty of Medicine and Health, University of Leeds, Leeds, United Kingdom; ^2^ Scleroderma Programme, NIHR Leeds Musculoskeletal Biomedical Research Centre, Leeds, United Kingdom; ^3^ Signal Transduction and Tumour Microenvironment Group, Leeds Institute of Cancer and Pathology, University of Leeds, Leeds, United Kingdom; ^4^ Cardiovascular Research Group, Faculty of Life Sciences, University of Bradford, Bradford, United Kingdom

**Keywords:** carbon monoxide, endothelial dysfunction, angiogenesis, systemic sclerosis, hemoxygenase-1, calcium homeostasis, Kv7.1 channels

## Abstract

Systemic sclerosis (SSc) is a terminal disease characterized by vasculopathy, tissue fibrosis, and autoimmunity. Although the exact etiology of SSc remains unknown, endothelial dysfunction, oxidative stress, and calcium handling dysregulation have been associated with a large number of SSc-related complications such as neointima formation, vasculogenesis, pulmonary arterial hypertension, impaired angiogenesis, and cardiac arrhythmias. Hemeoxygenase-1 (HO-1) is an antioxidant enzyme involved in multiple biological actions in the cardiovascular system including vascular tone, angiogenesis, cellular proliferation, apoptosis, and oxidative stress. The aim of this work was to investigate the physiological role of HO-1 and its relevance in the cardiovascular complications occurring in SSc. We found that, in early phases of SSc, the expression of HO-1 in dermal fibroblast is lower compared to those isolated from healthy control individuals. This is particularly relevant as reduction of the HO-1/CO signaling pathway is associated with endothelial dysfunction and vasculopathy. We show evidence of the role of HO-1/carbon monoxide (CO) signaling pathway in calcium handling. Using an *in vitro* model of pulmonary arterial hypertension (PAH) we investigated the role of HO-1 in Ca^2+^ mobilization from intracellular stores. Our results indicate that HO-1 regulates calcium release from intracellular stores of human pulmonary arterial endothelial cells. We interrogated the activity of HO-1 in angiogenesis using an organotypic co-culture of fibroblast-endothelial cell. Inhibition of HO-1 significantly reduced the ability of endothelial cells to form tubules. We further investigated if this could be associated with cell motility or migration of endothelial cells into the extracellular matrix synthesized by fibroblasts. By mean of holographic imaging, we studied the morphological and functional features of endothelial cells in the presence of an HO-1 activator and selective inhibitors. Our results demonstrate that inhibition of HO-1 significantly reduces cell proliferation and cell motility (migration) of cultured endothelial cells, whilst activation of HO-1 does not modify either morphology, proliferation or motility. In addition, we investigated the actions of CO on the Kv7.1 (KCQN1) channel current, an important component of the cardiac action potential repolarization. Using electrophysiology (whole-cell patch-clamp in a recombinant system overexpressing the KCQN1 channel), we assessed the regulation of KCQN1 by CO. CORM-2, a CO donor, significantly reduced the Kv7.1 current, suggesting that HO-1/CO signaling may play a role in the modulation of the cardiac action potential via regulation of this ion channel. In summary, our results indicate a clear link between: 1) downregulation of HO-1/CO signaling; and 2) pathophysiological processes occurring in early phases of SSc, such as calcium homeostasis dysregulation, impaired angiogenesis and cardiac arrhythmias. A better understanding of the canonical actions (mainly due to the biological actions of CO), and non-canonical actions of HO-1, as well as the interaction of HO-1/CO signaling with other gasotransmitters in SSc will contribute to the development of novel therapeutic approaches.

## Introduction

Systemic Sclerosis (SSc) or scleroderma is an immune-mediated disease characterised by vasculopathy and tissue fibrosis, the latter defined as pathological remodelling of connective tissue which leads to excessive deposition of collagen and other extracellular matrix proteins eventually affecting multiple organs, including the skin, lung, heart and gastrointestinal tract (Denton, 2015; Denton, 2016; López-Isac et al., 2019). SSc is an uncommon disease with high case-specific mortality: >50% of patients affected with SSc eventually die as a direct result of the disease. It is estimated that 2.7 million people worldwide have SSc. The incidence in adults aged ≥16 was 22.8 per million person-years (Mawdsley, 2006). A Canadian study estimated nearly 16,000 individuals suffer from scleroderma annually, with women being affected nearly 5 to 6 times more than men, with estimated direct health care costs of $18,453 per patient (Canadian Scleroderma Research Group, 2021). No cure currently exists for SSc, and therefore there is a high unmet medical need (Denton, 2020).

Endothelial cell (EC) damage is a key early process in SSc, which precedes fibrosis and involves phenotypic changes in ECs present in arterioles of skin microvasculature, where a population of ECs undergo endothelial-mesenchymal transition (EndoMT), leading to accumulation of myofibroblasts within the vessel wall and subsequent development of vasculopathy (Pattanaik et al., 2015; Romano et al., 2021). Vasculopathy is characterised by oxidative stress (OXS), inflammation, vasoreactivity, fibrotic intima hyperplasia, impaired neoangeogenesis with a gradual obliteration of vessels, and decline in capillary density (Abraham and Distler, 2007; Kahaleh, 2008; Ladak and Pope, 2016).

The early clinical signs of scleroderma vary, although Raynaud’s phenomenon (RP) and gastroesophageal reflux are cardinal clinical manifestations at this stage of the disease. Defective angiogenesis manifests in the form of drop out of capillaries and abnormal capillary architecture without a compensatory process. This leads to other vascular dysfunctions typically occurring in the onset of SSc such as digital ulcers (Korn et al., 2004), and nail-fold capillary abnormalities (Bissell et al., 2016). There are conflicting reports regarding the presence and role of circulating endothelial progenitor cells in SSc. However, it is well established that tissue fibrosis (promoted by aberrant TGF-β signaling) and immune dysregulation are key components involved in the systemic effects of the disease in later phases (i.e. presence of proliferative vasculopathy with intimal proliferation in peripheral, pulmonary, coronary, and renal arteries in the absence of inflammation is a hallmark feature of SSc).

Clinical and pathologic manifestations of SSc are always the result of three interactive processes: 1) innate/adaptive immune system abnormalities leading to production of autoantibodies and cell-mediated autoimmunity; 2) microvascular endothelial cell/small vessel fibroproliferative vasculopathy; and 3) fibroblast dysfunction generating excessive accumulation of collagen and other matrix components in skin and internal organs (Pattanaik et al., 2015; Denton and Khanna, 2017; Singh et al., 2019). All these processes affect each other, worsening the disease and leading to systemic clinical manifestations such as pulmonary arterial hypertension (PAH), cardiac arrhythmias and cardiac failure.

The main types of SSc are limited cutaneous (lcSSc) and diffuse cutaneous (dcSSc) scleroderma. lcSSc is defined by the presence of distal skin thickness, long history of RP and late-stage organ involvement leading to pulmonary arterial hypertension (PAH) and cardiac complications. dcSSc occurs with proximal limb/trunk involvement with skin sclerosis, short history of RP, increased risk of renal crisis and cardiac involvement, and high frequency of sever lung fibrosis. Other relevant subsets include *Sine Scleroderma* (SSc-related internal organ disease and RF, but lack of skin sclerosis) and *Overlap Syndrome SSc* (one of the three previous subsets together with another autoimmune rheumatic disease) (Denton and Khanna, 2017). In addition, pulmonary hypertension in SSc can manifest in three different forms: 1) Pulmonary Arterial Hypertension–vasculopathy of the small pulmonary arteries (group 1; PAH); 2) Myocardial fibrosis leading to left ventricular systolic or diastolic dysfunction (group 2: myocardial fibrosis); and 3) Interstitial Lung Disease–PH due to lung disease (group 3; ILD).

Arrhythmias, conduction system abnormalities, and direct myocardial disease (i.e. myositis, cardiac failure, cardiac fibrosis, and coronary artery diseases) are frequent cardiac manifestations in SSc (Veerman et al., 2013; Wu and Larsson, 2020). Primary myocardial manifestations result from the underlying vasculopathy and fibrosis that impair microcirculation and myocardial function (Kahan and Allanore, 2006). Arrhythmias and conduction abnormalities worsen the overall prognosis of SSc. These abnormalities may apparently be mild but can lead to a fatal outcome like sudden cardiac death in SSc (Ross et al., 2021). Although myocardial damage and fibrosis are clearly important factors leading to conduction abnormalities, the underlying arrhythmogenic mechanisms observed in SSc are not well understood, and the activity of cardiac ion channels responsible for the cardiac myocyte action potential have not been investigated in the context of SSc with cardiac involvement. Amiodarone is often used in the clinic as an effective antiarrhythmic drug, however anti-arrhythmic treatment must be adapted to the individual SSc patient (Vacca et al., 2014).

A very common electrocardiographic abnormality in SSc patients is QT-interval prolongation arrhythmias. Morelli and collaborators reported that 90% of SSc patients with conduction abnormalities showed QT-interval prolongation arrhythmias (Morelli et al., 1996); and this is currently a criterion to determine cardiac involvement in SSc patients. The QT-interval prolongation may arise from either a decrease in repolarizing cardiac membrane currents or an increase in depolarizing cardiac currents late in the cardiac cycle. Most commonly, QT-interval prolongation is produced by delayed repolarization due to a reduction in the delayed rectifier potassium current in cardiac action potentials. The two components of the delayed rectifier K^+^ current are the rapidly (IKr) and the slowly (IKs) activating delayed repolarizing cardiac K^+^ current. KCNH2 gene encodes the α-subunits of the K^+^ channel that conducts the cardiac rapid delayed rectifier K^+^ current (IKr, Kv11.1 channel) and KCNQ1 gene encodes the α-subunits that co-assemble to form the K^+^ channel that conducts the cardiac slow delayed rectifier K^+^ current (IKs, Kv7.1 channel). (Sanguinetti and Zou, 1997; Viswanathan et al., 1999; Tamargo et al., 2004; Moss and Kass, 2005; Chiamvimonvat et al., 2017). A reduction of I_Kr_ is associated with ventricular arrhythmia and in some cases (i.e. long QT syndrome (LQTS)-associated mutations in KCNH2) can cause sudden cardiac death (Schwarz and Bauer, 2004; Sanguinetti, 2010). Remarkably, SSc patient are at higher risk of sudden cardiac death. However, the role of Kv7.1 and Kv11.1 channels have not been investigated in SSc.

Hemoxygenase-1 (HO-1) is an inducible enzyme with antioxidant properties that is expressed in response to environmental stress agents (such as heavy metals and hypoxia) and endogenous mediators (NO, heme and cytokines). HO-1 controls vascular tone, angiogenesis, cellular proliferation, apoptosis and oxidative stress (OXS) via its catalytic products, ferrous iron (Fe^2+^), biliverdin, and carbon monoxide (CO) (Ryter and Choi, 2009). Although Fe^2+^ and biliverdin are involved in the management of redox homeostasis, here we focus on the physiological actions of CO, since it is well established that CO exerts antioxidant, anti-proliferative and anti-inflammatory actions by influencing multiple cellular pathways (Araujo et al., 2012; Dennery, 2014). In addition, induction of HO-1 protects EC cells from OXS, and this prevents endothelial dysfunction (Durante, 2010; Calay and Mason, 2014; Durante, 2020). Importantly, TGF-β (the main pro-fibrotic factor in SSc) suppresses the expression of HO-1 by displacing the nuclear erythroid-derived 2 (Nrf-2) transcription factor from the promoter of HO-1 (Okita et al., 2013), however this mechanism has not been explored either in early phases of SSc nor in SSc-PAH.

In the cardiovascular system, CO is a potent endogenous physiological modulator of vascular tone, cellular proliferation, apoptosis, mitochondrial function, redox homeostasis, immune response and inflammation (Brouard et al., 2000; Ndisang et al., 2004; Durante et al., 2006; Desmard et al., 2007; Wegiel et al., 2014; Duckles et al., 2015; Loboda et al., 2016; Oliveira et al., 2016; Durante, 2020; Chen et al., 2021). In addition, CO inhibits platelet aggregation via inhibition of cytochrome c oxidase (Chlopicki et al., 2012; Kaczara et al., 2020), and promotes myocardial cell differentiation and maturation via redox regulation of mitochondria biogenesis (Suliman et al., 2016).

As opposed to other gasotransmitters, CO does not interact covalently with amino acids or major cellular components, except for preferential reactivity towards selected transition metals in a specific redox state which are present in cellular proteins, including ferrous heme-dependent proteins (Fukuto et al., 2012; Motterlini and Foresti, 2017). Some of the cellular targets responsible for the biological effects of CO include p38 mitogen-activated protein kinases (p38MAPK), peroxisome proliferator activator-gamma (PPARγ), signal transduction and activator of transcription-3 (STAT-3), phosphatidylinositol-3- kinase/Akt (PI3K/Akt), and hypoxia-inducible factor-1 (HIF-1) (Otterbein et al., 2003; Zhang et al., 2006; Wang et al., 2007; Hoetzel et al., 2008; Roos et al., 2011).

Other cellular targets which are often regulated by CO (and other gasotransmitters) are ion channels (Peers et al., 2009; Wilkinson and Kemp, 2011; Peers et al., 2015). For instance, regulation of Ca^2+^-dependent K^+^ (BK_Ca_) channels by CO is physiologically relevant in vasodilation and control O_2_ sensing by carotid body chemoreceptors (Wang and Wu, 1997; Williams et al., 2004); inhibition of the cardiac voltage-gated Na^+^ channel (Na_v_1.5) by CO can lead to cardiac arrhythmias (Dallas et al., 2012; Elies et al., 2014); inhibition of cardiac myocyte L-type Ca^2+^ currents (Ca_v_1.2 channels) is mediated by CO-induced increases in mitochondrial reactive oxygen species (ROS) (Scragg et al., 2008); and the CO-mediated inhibition of T-type Ca^2+^ (Ca_v_3.1, 3.2, and 3.3) channels in the vasculature plays an important role in smooth muscle cell proliferation (Boycott et al., 2013; Duckles et al., 2014; Duckles et al., 2015). CO increases vasodilation in the microvascular circulation and is currently being investigated as a therapeutic approach in PAH and preeclampsia (Guohua et al., 2003; Stanford et al., 2004; McRae et al., 2019).

There is ample evidence supporting the protective role of HO-1/CO system in the development and progression of PAH (Morita et al., 1995; Christou et al., 2000; Guohua et al., 2003; Durante, 2020). Importantly, several *in vivo* studies reported that HO-1 induction (or CO inhalation) prevents the development of PAH (Minamino et al., 2001; Shimzu et al., 2008). In contrast, inhibition of HO-1 activity by ZnPP increases mean pulmonary arterial pressure, the medial thickness and muscularization of pulmonary arteries in a rat chronic hypoxia model of PAH (Gong et al., 2004).

Ca^2+^ oscillations play a central role in the regulation of gene expression in human vasculature (Janssen et al., 2015). However, the mechanisms by which cytosolic [Ca^2+^] are regulated and [Ca^2+^] oscillations transduced are both poorly understood in the context of SSc. In addition, the use of vasodilatory agents and calcium blockers as regular drugs for the management of the clinical symptoms in early phases of SSc, clearly indicates that dysregulation of calcium homeostasis is a hallmark of the disease.

Since cellular calcium homeostasis and angiogenesis are two endothelial cell functions which are impaired in early stages of SSc, we chose to investigate the role of HO-1 in endothelial function in the context of intracellular calcium handling and neoangiogenesis. In addition, since a decrease in the Kv7.1 current is associated with QT-interval prolongation arrhythmias, and this is a common cardiac conduction abnormality in established cardiac involvement SSc (late phases of the disease), we studied the effects of CO in recombinant Kv7.1 current, an important component of the cardiac action potential repolarization. Our results show that HO-1 plays a role in Ca^2+^ homeostasis and promotes angiogenesis in SSc, suggesting that promotion of HO-1/CO signaling in early phases of the disease may be a potential therapeutic approach to prevent endothelial dysfunction. On the other hand, CO inhibits Kv7.1 K^+^ channel conductance, indicating that modulation of HO-1/CO signaling could be targeted as a novel therapeutic target to treat QT-interval prolongation arrhythmias in late phases of SSc.

## Materials and Methods

### Cell Culture and Culture Treatments

Human lung microvascular endothelial cells, HLMECs (Lonza; cc-2543), were cultured in EGM™-2MV microvascular EC growth Medium-2 (Lonza; cc-3202) containing 5% FBS under normoxic (20% O_2_) and hypoxic (1% O_2_ (balanced N_2_), 5% CO_2_) conditions. The hypoxic condition maintained for 24 h was used as an *in vitro* model to mimic chronic hypoxia (CH), and an *in vitro* model of pulmonary arterial hypertension. Human control HC and SSc dermal fibroblasts: skin biopsies from 5 patients with early limited cutaneous systemic sclerosis (lcSSc) and five healthy controls were obtained from the Scleroderma clinic within the Leeds Institute of Rheumatic and Musculoskeletal Medicine (United Kingdom). Biopsies were taken with full informed consent as approved by National Research Ethics Service (NRES) Committee (REC 10/H1306/88) and processed as described previously in Liakouli and co-workers (Liakouli et al., 2018). Human umbilical vein endothelial cells (HUVECs) were obtained from Caltag Medsystems (TCS Cellworks), cultured in fully supplemented human large vessel endothelial cell medium (TCS Cellworks, United Kingdom) and only used for experiments at passages between 2 and 4. To evaluate the effect of TGF-β on HO-1 expression, cells were grown to confluence in six-well culture plates in Dulbecco modified eagle medium (DMEM) 10% fetal calf serum (FCS), serum starved in DMEM 0.5% FCS for 24 h and stimulated in the presence of 10 ng/ml recombinant human TGF-β1 (Sigma, United states) for 48 h.

### Western Blotting

Western blotting was used to confirm the expression of HO-1 in dermal fibroblasts and HUVEC cells. In brief, proteins were separated on 12% SDS-PAGE gels using a BioRad Mini Protean II electrophoresis system (Bio-Rad, United states). Separated proteins were then transferred onto poly-vinylidene difluoride membranes, and the blots incubated in 3% (w/v) non-fat dried milk in Tris-buffered saline (TBS) prior to probing with anti-HO-1 mouse monoclonal antibody (Invitrogen, #MA1-112) or mouse anti-Human HO-1 [1F12-A6] monoclonal antibody (StressMarq Biosciences, #SMC-131) at 1:1,000 dilution in 0.3% (w/v) BSA in TBS for 16 h at 4°C. Approximate equal loading of total protein (30 µg per lane) was confirmed by blotting with monoclonal β-actin antibody (1:5,000 Sigma, St Louis, MO, United states) in 0.3% (w/v) BSA in TBS for 16 h at 4°C. After three 5-min washes with TBS, membranes were probed with 1:2000 dilution of horseradish peroxidise labelled rabbit anti-mouse immuno-globulin G secondary antibody (Sigma, # 31194) in 0.1% (w/v) BSA in TBS for 1 h at 20°C. Following three 5-min washes with TBS, enhanced chemiluminescence (ECL) SuperSignal West Pico substrate solution (Pierce, Rockford, IL, United states) was used to generate the chemiluminescent signal, which was detected using a BioRad ChemiDoc XRS imaging system. Intensity of the bands was calculated using ImageJ software. The intensity of each HO-1 band was normalized by the intensity of the corresponding β-actin band.

### Measurement of [Ca^2+^]_i_

Cells were grown on glass coverslips in 6-well culture plates to approximately 60% confluence and then conditioned under normoxia (24 h, 20% O_2_) or chronic hypoxia (24 h, 2.5% O_2_). On the day of the experiment, cells were incubated with perfusate buffer (NaCl 135 mM, KCl 5 mM, MgSO_4_ 1.2 mM, CaCl_2_ 2.5 mM, HEPES 5 mM and glucose 10 mM; pH 7.4, osmolarity adjusted to 300 mOsm with sucrose) containing 4 µM Fura-2AM (Invitrogen, F1221) for 40 min at 37°C in the dark, then left to de-esterify for 15 min in control solution alone. Fragments of cell-populated coverslips were then transferred into an 80 µl recording chamber mounted on the stage of an inverted microscope and perfused under gravity at a rate of ca. 5 ml/min. Ca^2+^-free perfusate contained 1 mM EGTA and no added Ca^2+^. [Ca^2+^]_i_ was determined ratiometrically using a *Cairn Research multiple excitation dual emission photometry system* (Optoscan, Cairn Research Ltd., United Kingdom); alternating excitation 340 and 380 nm (0.2 Hz), emission 510 nm. Regions of interest were selected to restrict data collection to individual cells. Drugs employed to probe Ca^2+^ homeostasis were added to the perfusate as indicated in the results. Data are presented as ‘raw’ (uncalibrated) ratio units rather than converted to molar [Ca^2+^] since calibration, while useful in many ways, can introduce errors and so actual molar values can be misleading (Becker and Fay 1987; Fowler and Tiger 1997).

### Organotypic Angiogenesis Assays

The organotypic co-culture assay (Mavria et al., 2006; Hetheridge et al., 2011) was performed with HUVEC and either hTERT immortalised HC fibroblasts or SSc fibroblasts. Pharmacological inducers and inhibitors of HO-1 were applied 7 days after initiation of the coculture with no change of media for 48 h prior to application. Tube formation was assessed by immunohistochemistry using a mouse anti-human CD31 Tubule Staining Kit (TCS CellWorks). Anti-human CD31 was applied at 1:400 dilution and alkaline phosphatase-conjugated secondary at 1:500. The substrate was applied for 15 min and washed. Digital images of the cocultures were taken immediately after staining. Organotypic coculture assays of HUVEC with human dermal fibroblasts were set up as previously described (Bishop, 1999). Number of tubules and total tubule length were analysed using the AngioSys 2.0 software (TCS Cellworks, Buckingham, United Kingdom).

### Live Holographic Imaging

Holographic microscopy analysis was performed to investigate the morphological and functional changes caused by inducers and inhibitors of HO-1 in endothelial cells. Holographic imaging recorded in real-time was used to monitor morphological (cell area, optical thickness, and optical volume) and functional (cell motility, cell tracking, and cell migration) parameters using the Holomonitor App Suite (Phase Holographic Imaging PHI AB). HUVECs were seeded at low confluency (100 k cells per well) onto 6-well plates (Sarstedt, Germany). Drugs and VEGF were added to the media at the concentration indicated in Figure 4 (25 ng/ml VEGF, 10 µM CoPPIX, and 1 µM ZnPPIX). For every independent experiment, one control well (absence of drugs or VEGF) was included in each plate. The plate was placed onto the xy motorised stage of a HoloMonitor M4 Microscope, set up inside an incubator using Phi HoloLids™ imaging covers (PHI, phase holographic imaging), previously sterilised in 70% EtOH, to optimise the acquisition quality of the holographic images. After automatic calibration of the background and microscope objective, a minimum of three fields/coordinates per well, randomly assigned by the software, were focused on. Holographic images were recorded at an interval of 5 min over 48 h using the HoloMonitor M4 (Phase Holographic Imaging PHI AB, Lund, Sweden) inside the cell-culture incubator (37°C, 5% CO_2_). Post-acquisition, the images were analysed applying the “Otsu mask”, where a threshold was set to distinguish cells from the bottom of the well (cell segmentation), allowing for automatic cell identification. Automatic cell number assignment allowed for individual cell tracking over time, which was double-checked manually as described in (Owston et al., 2019). All outputs were exported into Excel files and plotted using Prism 9 (GraphPad).

### Electrophysiology

Whole-cell potassium currents were recorded with an Axopatch 200 A amplifier (Molecular Devices) from cells bathed in a perfusate comprising, in mM: 130 NaCl, 4 KCl, 1.2 MgCl_2_, 2 CaCl_2_, and 10 HEPES; pH was adjusted to 7.4 with NaOH. Electrodes were fabricated from borosilicate glass and filled with a filtered solution containing, in mM: 130 KCl, 1 MgCl_2_, 5 EGTA, 5 K_2_ATP, and 10 HEPES; pH was adjusted to 7.2 with KOH. Electrode resistances were 2–4 MΩ and the mean whole-cell capacitance was 14 ± 0.31 pF. Currents were evoked with 2-s test pulses from −80 to 80 mV from a holding potential of −80 mV, with 20 mV increments. Tail currents were recorded at −40 mV for 2.5 s. The interpulse interval was 5 s. Relative current was calculated from the tail currents and expressed as pA/pF as a function of the membrane voltage. All electrophysiological data were analysed in pClamp11 (Molecular Devices), Excel, and Prism 9 (GraphPad).

### Statistical Analysis

Data were analysed with GraphPad Prism 9.0 (GraphPad Software, San Diego, United states) and expressed as means ± SEM from at least three independent experiments (unless otherwise indicated). Comparisons between groups were performed with Kruskal–Wallis’ nonparametric analysis of variance test followed by two-by-two comparisons with Mann-Whitney’s *U* test when a significant difference was estimated. *p* < 0.05 was considered statistically significant. For Ca^2+^ fluorometry experiments, statistical comparisons were made using unpaired Student’s t-tests. All mean data were obtained from cell populations of at least 8 individual cells from 3 independent experiments.

## Results

**FIGURE 1:**
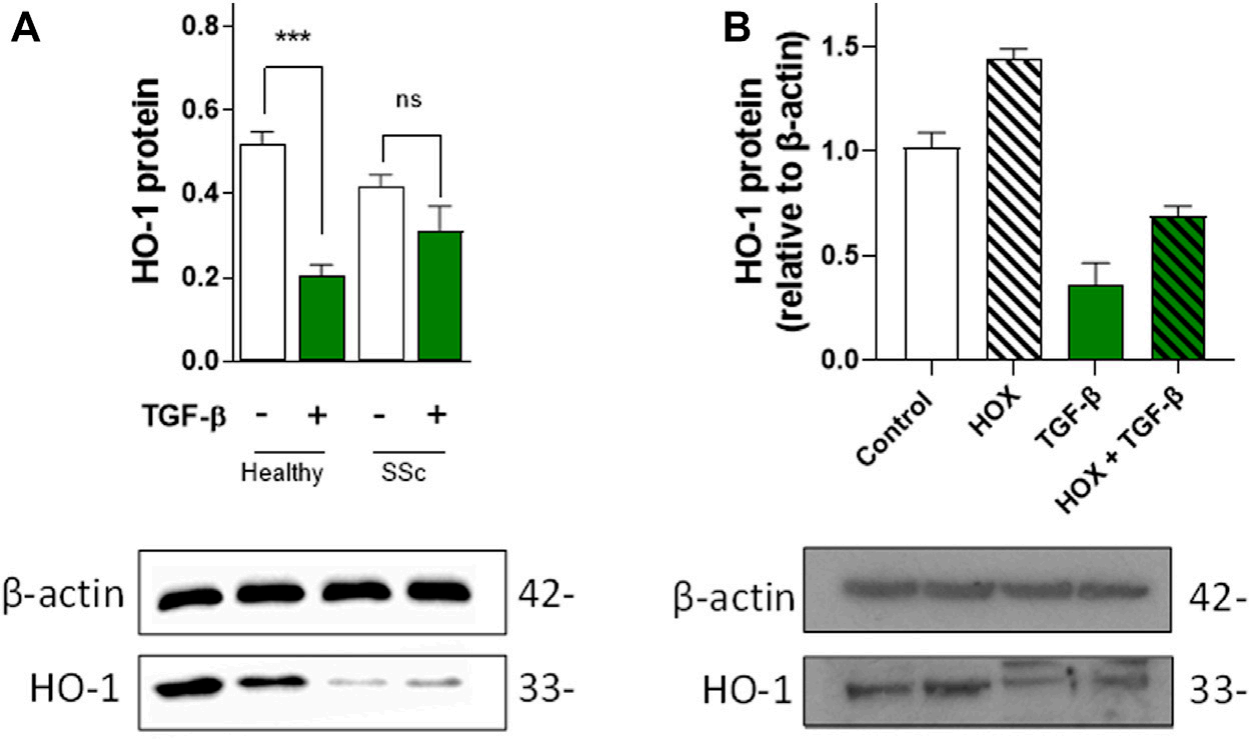
Effects of TGF-β on HO-1 expression in dermal fibroblasts and vascular endothelial cells. **(A)**. TGF-β decreases HO-1 expression in healthy control fibroblast but not in SSc fibroblasts. Western blot data (n = 5). **(B)**. TGF-β decreases HO-1 expression and prevents hypoxia (HOX)-dependent induction in HUVEC (n = 4).

As fibroblasts are responsible for the production and secretion of TGF-β (the main pro-fibrotic factor in SSc), we investigated the expression levels of HO-1 in healthy and SSc fibroblasts. Although expression levels of HO-1 in unstimulated SSc fibroblasts are not significantly different from healthy fibroblasts, exposure to 10 µM TGF-β for 24 h significantly inhibited the expression of HO-1 in healthy but not SSc fibroblasts (Figure 1A), indicating that the expression of HO-1 is already influenced by the elevated endogenous production of TGF-β in SSc fibroblasts.

Since aberrant TGF-β signaling can inhibit the expression of HO-1 (Okita et al., 2013), we decided to investigate the paracrine effects of TGF-β in endothelial HO-1 expression under chronic hypoxia (HOX, 1% O_2_ for 24 h; a well-established *in vitro* model of PAH) (Aley et al., 2008) and normoxia conditions (Control, 20% O_2_). Exogenous TGF-β in vascular endothelial cells (HUVEC) not only inhibited HO-1 expression but also blocked chronic hypoxia-dependent induction of HO-1 (Figure 1B). These results indicate that HO-1 expression is inhibited by TGF-β in EC and that this downregulation might be a primary cause of endothelial dysfunction.

**FIGURE 2:**
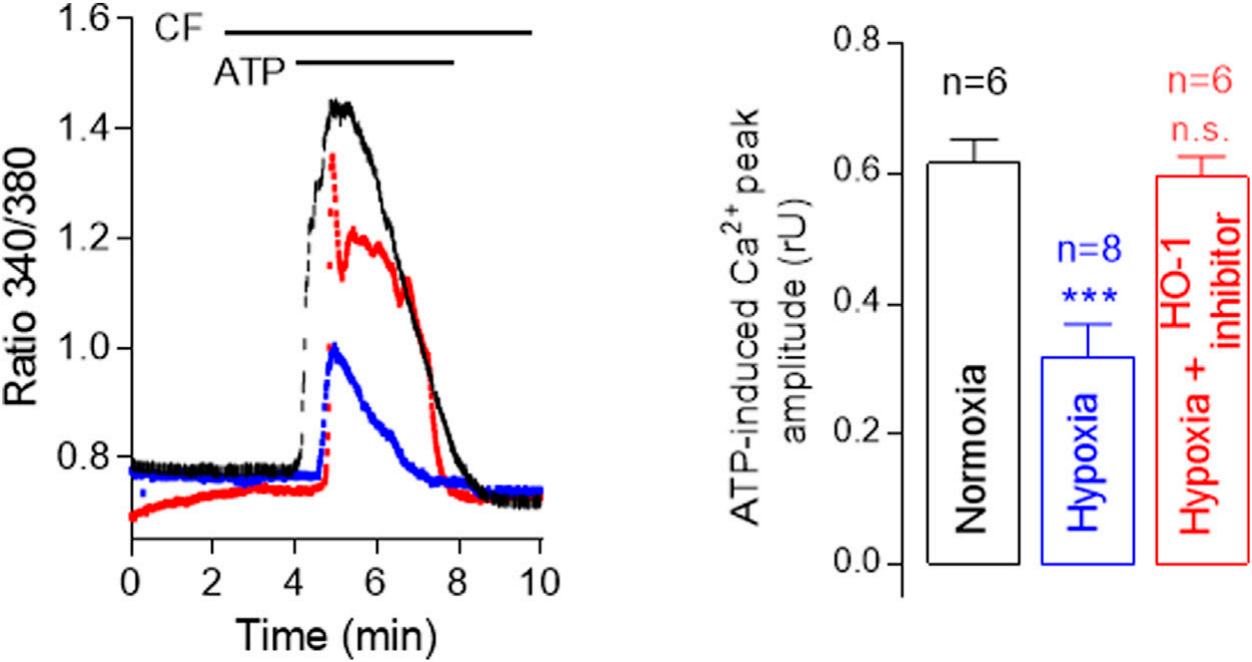
Hypoxia decreases ATP-induced [Ca2+]i increase in human pulmonary microvascular endothelial cells (HPMVECs). Representative traces showing ATP-induced Ca^2+^ increase in calcium-free (CF) solution using Fura-2 AM of control (Normoxia, 20% O_2_, black), Hypoxia (blue) and Hypoxia + HO-1 inhibitor, 15 µM QC-15 (red). Note that Ca^2+^ release from intracellular stores is significantly lower under hypoxia, and this effect is reversed by the inhibition of HO-1 activity.

Moreover, chronic hypoxia causes Ca^2+^-dependent morphological changes in pulmonary arteries, such as neomuscularization, wall thickening, and angiogenesis (Howell et al., 2003; Wade et al., 2018) and is known to increase HO-1 expression. Taking this into account, we sought to investigate the role of HO-1 in calcium mobilization from intracellular stores in human pulmonary microvascular endothelial cells (hPMVEC). Our results show that chronic hypoxia decreases ATP-induced [Ca^2+^]_i_ increase in human pulmonary microvascular EC (Figure 2). This effect is attenuated by the selective HO-1 inhibitor QC-15, indicating that HO-1 influences intracellular Ca^2+^ signaling.

In a previous report we showed that tubule formation of HUVEC or human microvascular endothelial cells (MVEC) is impaired when cells are co-cultured with SSc fibroblasts compared to healthy fibroblasts (Liakouli et al., 2018). We demonstrated that the anti-angiogenic effect is mediated by the secretion of the serpin pigmented epithelium-derived factor (PEDF) and depends on the aberrant TGF-β signaling.

**FIGURE 3:**
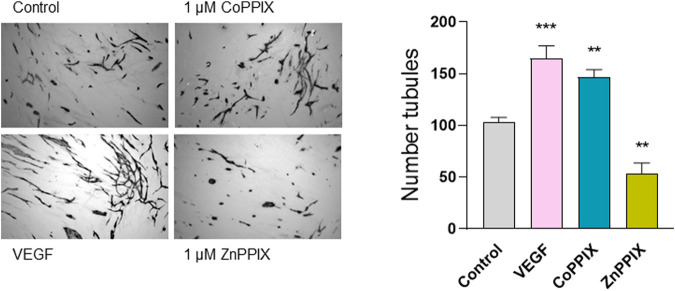
HO-1 induction promotes (whilst HO-1 inhibition decreases) tubule formation in organotypic model of angiogenesis. Images represent microscopic fields from co-cultures assays of human umbilical vascular endothelial cells (HUVECs) seeded onto confluent dermal fibroblasts (FB) isolated from SSc patients in the absence of any treatment (Control), 1 µM CoPPIX (HO-1 induction), and 1 µM ZnPPIX (HO-1 inhibitor), and stained for the endothelial marker CD31 (FBs are seen unstained in the background). 25 ng/ml VEGF was used as a positive control. Histograms show the number of tubules using AngioSys 2.0 (TCS Cellworks, Buckingham, United Kingdom),represented as mean ± SEM (n = 10 microscopic fields at 4x magnification from triplicate wells).

To determine whether the observed decreased in HO-1 expression induced by aberrant TGF-β signaling contribute to defective angiogenesis, we performed endothelial-fibroblasts organotypic angiogenesis *in vitro* assays in which endothelial cells form tubules highly reminiscent of capillaries formed during angiogenesis *in vivo*, embedded in a natural matrix produced by SSc fibroblasts. Induction of HO-1 with CoPPIX (1 µM, 48 h) significantly increased the number of tubules (50% increase compared to control, absence of treatment). On the contrary, inhibition of HO-1 decreased the number of tubules formed under the same conditions (40% decrease compared to control). These results indicate that HO-1 plays a role in the tubule formation in the context of SSc (Figure 3).

**FIGURE 4:**
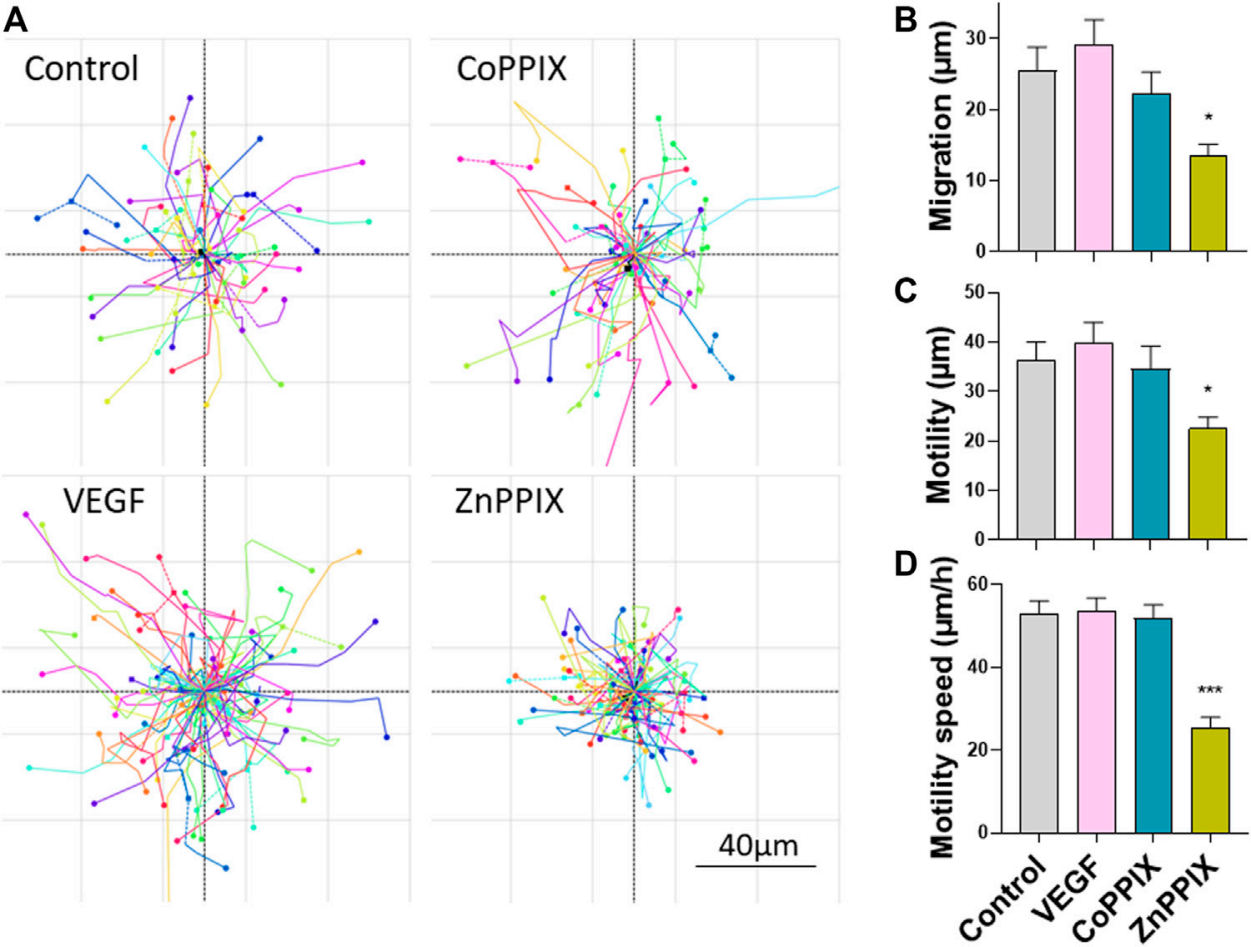
Inhibition of HO-1 decreases migration, motility and motility speed of HUVEC cells. **(A)**. Images represent migration plots of HUVECs in culture conditions in the absence of any treatment (Control), 1 µM CoPPIX (HO-1 induction), 1 µM ZnPPIX (HO-1 inhibitor), and 25 ng/ml VEGF. Each migration plot shows the total distance (µm) of the trail completed by each cell present in the field of view in a specific coordinate for a period of 30 min after 24 h of treatments. Histograms represent mean ± SEM of migration **(B)**, motility **(C)** and motility speed **(D)** for the conditions indicated above (n = 3 independent repeats; each repeat includes data from 3 coordinates per experimental condition).

Considering that HO-1 activity promotes angiogenesis, we sought to investigate whether the proangiogenic effects of HO-1 are directly influencing endothelial function (only HUVECs) or, alternatively, the proangiogenic effects of HO-1 rely on the presence of FBs. By means of holographic imaging we studied the morphology, proliferation, migration and motility of cultured HUVECs for a period of 48 h in the presence of HO-1 inducers and inhibitors (Figure 4). Our results indicate that induction of HO-1 did not significantly affect morphology, proliferation nor motility of HUVEC compared to Control (absence of treatment) or 25 ng/ml VEGF. However, inhibition of HO-1 (1 µM ZnPPIX), although did not affect cell morphology, induced a significant decreased in cell proliferation (data not shown), migration and motility speed in HUVECs (Figure 4).

**FIGURE 5:**
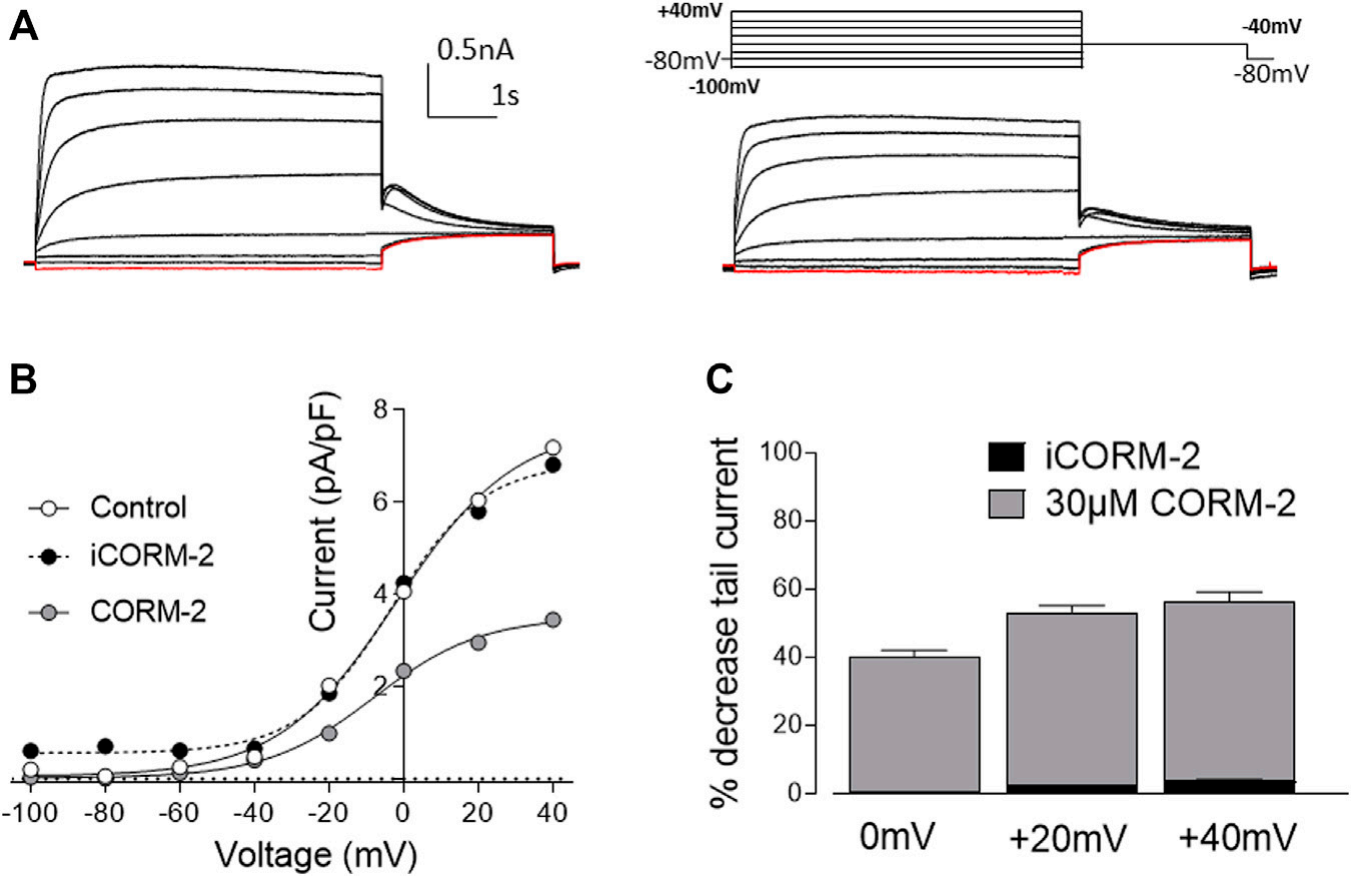
Carbon monoxide inhibits Kv7.1 currents. **(A)**. Representative traces of Kv7.1 current elicited by a family of pre-pulses ranging from -100mV to +40mV for 5s in the absence (left) and presence (right) of CORM-2. Tail current was evoked at -40mV for a period of 2.5s. Note the decrease of the tail current in the presence of CORM-2. **(B)**. I-V relationship of the tail current in the absence (white circles) and presence (grey circles) of CORM-2. Note that the application of iCORM-2 (compound without CO) did not elicit significant changes in the magnitude of the tail current. **(C)**. Histogram represents percentage of decrease of the tail current compared to control for CORM-2 (grey) and iCORM-2 (black) at 0, 20 and 40 mV. Data expressed as mean ± SEM (n = 7 cells).

One of the main biological activities of CO is the regulation of ion channels via multiple signaling pathways (Peers et al., 2015). We have previously demonstrated that CO modulates the activity of cardiac ion channels. For instance, L-type Ca^2+^ channels are inhibited by CO via mitochondrial ROS formation (Scragg et al., 2008), whereas augmentation of late cardiac sodium current (I_NaL_) by CO required NO formation (Dallas et al., 2012), and the inhibition of the cardiac rapid delayed rectifier K^+^ current (I_Kr_) is mediated by peroxynatrite (Al-Owais et al., 2017). Another important component of the cardiac repolarization action potential is the slow delayed rectifier K^+^ current (I_Ks_) which is elicited by the K^+^ efflux through Kv7.1 ion channels. A decrease in Kv7.1 conduction is a plausible cause for QT-interval prolongation arrhythmias, and since this type of arrhythmia is common in SSc, we investigated the effects of CO on recombinant Kv7.1 channels by means of whole-cell patch-clamp electrophysiology. 30 µM CORM-2 (CO donor) elicited a decrease of the Kv7.1 tail current (approximately 50% reduction at 20 mV and 60% at 40 mV), whilst iCORM-2 did not show an effect on the ion channel current (Figure 5).

## Discussion

This study reports for first time a decrease on the expression of HO-1 in dermal fibroblasts isolated from early limited cutaneous SSc (lcSSc). In addition, a decrease in the expression of HO-1 in endothelial cells upon increased TGF-β signaling, indicates that in the context of early phases of SSc, induction of endothelial HO-1 is impaired. These results agree with previous work reported by Okita and collaborators (Okita et al., 2013) which demonstrated that aberrant TGF-β signaling inhibits HO-1 induction via promotion of the transcription factors MafK and Bach1, leading to the displacement of Nrf-2 from the HO-1 gene promoter and the subsequent suppression of HO-1 gene expression.

In addition, we report several novel effects of HO-1/CO signaling on Ca^2+^ homeostasis in human pulmonary aortic ECs, and angiogenesis in the context of SSc. Under chronic hypoxia, ATP-evoked Ca^2+^ released from intracellular stores was significantly reduced (compared to Ca^2+^ released from intracellular stores under normoxia), an effect which was almost completely reversed by the selective HO-1 inhibitor (15 µM QC-15). These results suggest that HO-1 plays a role in modulating Ca^2+^ buffering from the ER (and mitochondria) and could be linked to the protective actions of HO-1 in protecting endothelial cell function. It is well established that Ca^2+^ overload (increased of Ca^2+^ buffering in the ER or mitochondria) in vascular and cardiac cells is associated with several cardiovascular conditions such as heart failure (Korf-Klingebiel et al., 2021), ischemia-related dysfunction, and pulmonary arterial hypertension (Suresh and Shimoda, 2017). Previous reports showed that chronic hypoxia can profoundly modulate Ca^2+^ homeostasis of smooth muscle cells (Wang et al., 2006; Aley et al., 2008), however this mechanism has not been investigated in the context of SSc.

Saliently, the initiation of endothelial dysfunction occurring in PAH, by which a particular population of ECs undergo EndoMT and become resistant to apoptosis (Good et al., 2015; Gorelova et al., 2021), resembles the same events described regarding ECs dysfunction in skin microvasculature in the early stages of SSc (Manetti et al., 2017; Romano et al., 2021). This suggests that the molecular changes occurring in endothelial cells from skin vasculature in the early stages of SSc are critical to the pathogenesis of PAH associated with SSc (SSc-PAH), and potentially preventing these events could stop the subsequent development of PAH. It is therefore critical to understand the molecular mechanisms of endothelial dysfunction to develop novel therapeutic approaches focused on reversing vasculopathy and arresting the systemic effects in the lung microvasculature in late onset of SSc. Ca^2+^ homeostasis plays an important role in gene expression and changes in Ca^2+^ homeostasis might be linked to EndoMT. Further studies aiming to determine a link between Ca^2+^ homeostasis and EndoMT in SSc will shed light on the initial molecular processes which occur in early phases of the disease and will provide an insight into novel therapeutic approaches to prevent endothelial disfunction.

We have reported that CO exposure causes early afterdepolarization arrhythmias, which correlate with QT-interval elongation (LQTS). In previous studies we showed that toxic environmental concentrations of CO cause cardiac arrhythmias as a result of augmentation of the late Na^+^ current (Dallas et al., 2012; Elies et al., 2014). In addition, CO induces arrhythmias in guinea pig cardiac myocytes via the peroxynitrite (ONOO^−^)-mediated inhibition of Kv11.1 K^+^ or hERG (KCNH2) channels (Al-Owais et al., 2017). The latter work showed that supramaximal (environmentally toxic) levels of exogenous CO cause early afterdepolarization arrhythmias in guinea pig cardiomyocytes. Action potentials (APs) from guinea pig cardiomyocytes more closely resemble those of human cardiomyocytes than does the rat cardiomyocyte, via inhibition of Kv11.1 K^+^ or hERG (KCNH2) channels.

In this study we identified the Kv7.1 K^+^ channel (the cardiac slow delayed rectifier K^+^ current, I_Ks_) as a novel molecular target for CO, by investigating the effect of CO perfusate or CORM-2 (CO donor) in a recombinant system of KCNQ1 (the α-subunit of the Kv7.1 channel). Vascular Kv7 channels control intracellular Ca^2+^ dynamics in smooth muscle (Tsai et al., 2020) and endothelial cells (Baldwin et al., 2020). In cardiac tissue, Kv7.1 are responsible for the cardiac slow delayed rectifier K^+^ current where they play an essential role in maintaining the duration of the cardiac AP.

Less commonly, QT interval prolongation results from prolonged depolarization due to a small persistent inward “leak” in cardiac sodium (Na^+^) current, also called late Na^+^ current, I_Na,L_ (Bennett et al., 1995). Importantly, CO gas exposure (or application of CORM-2) in the extracellular bath, induces proarrhythmogenic effects in ventricular cardiac myocytes via increased of the I_Na,L_ (Dallas et al., 2012; Elies et al., 2014). Whether this is a mechanism relevant in SSc is still to be determined.

Here we propose that the endothelial dysfunction observed in early phases of SSc is associated with the cellular inability to induce HO-1/CO signaling. This renders endothelial cell vulnerability, upon fibrosis and oxidative stress, which leads to vasculopathy. Tissue fibrosis (associated with aberrant TGF-β signaling) and immune dysregulation worsen the disease and contribute to maladaptive phenotypic changes, which in later phases of the disease, might lead to an increased in HO-1 expression (and increased CO signaling). Maladaptive cellular changes associated with an increase in the expression of HO-1 is observed in several forms of cancer (Cesna et al., 2019; Hemmati et al., 2021). In cardiac tissue, this enhancement in HO-1/CO signaling may be sufficient to trigger the pro-arrhythmogenic effects of CO via inhibition of Kv11.1 (Al-Owais et al., 2017) and inhibition of Kv7.1 channels.

It is noteworthy that cardiopulmonary involvement is the leading cause of SSc-related deaths (accounting for 73% of the total). In this work we provide preliminary observations which suggest that potentiation of HO-1/CO signaling in the early phases of the disease might contribute to prevention of endothelial dysfunction in terms of Ca^2+^ homeostasis and angiogenesis.

On the other hand, our results regarding inhibition of the cardiac Kv7.1 current by CO also indicate that an increase in HO-1/CO signaling might contribute to QT-interval elongation arrhythmias, suggesting that a potentiation of HO-1/CO signaling would be detrimental (with fatal consequences) in late phases of the disease when cardiac involvement has been established.

This discrepancy in the actions of HO-1 (or CO) has been also described in other pathophysiological scenarios such as vascular inflammation and atherosclerosis (Stocker and Perrella, 2006), cerebral ischaemia (Matsuoka et al., 1999; Terraneo and Samaja, 2017) and cancer (Szabo, 2016). In fact, it is recognized that CO (and other gasotransmitters) exhibits a bell-shaped (or bimodal) pharmacological character. This implies that low concentrations of CO (or NO/H_2_S) that are produced endogenously by HO enzymes (or gasotransmitter generating enzymes) can support cardioprotective effects of CO, whilst higher concentrations of CO can be detrimental (cytostatic or cytotoxic).

In any case, further studies focused on the determination of HO-1 expression levels in cardiac tissue in advanced phases of SSc need to be conducted, as well as the investigation of the effects of CO in endogenous cardiac Kv7.1 where it is co-expressed with the auxiliary subunit KCNE1 and KCNE2 (Plant et al., 2014).

The experimental work undertaken has focused on HO-1 and/or CO signaling, with other gasotransmitters like NO or H_2_S being out of scope for the current study. A substantial body of literature, however, conveys the beneficial actions of NO and H_2_S in SSc. For instance, the therapeutic potential of NO and H_2_S has been extensively reviewed (Cotton et al., 1999; Matucci Cerinic, 2002; Dooley et al., 2006; Malerba et al., 2007; Abdulle et al., 2018). Since the three gasotransmitter pathways interact, in order to better understand the pathophysiology of SSc, it is critical for future investigation to resolve potential levels and patterns of pathway crosstalk. Such studies could then help pave the way to novel therapeutic approaches aimed at preserving and restoring endothelial health.

## Data Availability

The original contributions presented in the study are included in the article/Supplementary Material. The datasets generated during and/or analyzed during the current study are available from the corresponding author upon reasonable request
